# Complete mitochondrial genome analysis of *Leptomias* sp. (Coleoptera, Curculionidae) from Southeast Tibet of China

**DOI:** 10.1080/23802359.2020.1797550

**Published:** 2020-07-25

**Authors:** Dong Xiang, Deqing Zhuoga, Wang Zhen, Jiancheng Zang

**Affiliations:** a Institute of Vegetable, Tibet Academy of Agricultural and Animal Husbandry Sciences, Lhasa, P. R. China; b Plant Science College, Tibet Agriculture and Animal Husbandry University, Nyingchi, P. R. China

**Keywords:** Mitochondrial genome, *Leptomias* sp, phylogenetic analysis

## Abstract

*Leptomias* sp. belongs to Coleoptera, Curculionidae, Leptomias Faust. It is mainly distributed in the Himalayas and Yarlung Zangbo River valleys in Tibet. It is the first time to report the complete mitochondrial genome of *Leptomias* sp., which featured a typical circular molecule of 16,801 bp in length, including 13 PCGs, 22 tRNAs, two rRNAs, and one control region. The overall nucleotide composition was 38.7% of A, 10.0% of G, 34.3% of T, and 17.0% of C. The content of A + T was higher than G + C. In this study, we also determined that *Leptomias* sp. was the sister to the *Sympiezomias velatus* based the phylogenetic analysis of nucleotide sequence datasets. This study provides a useful resource for further studies on conservation and population genetics of *Leptomias* species.

*Leptomias* sp. belongs to Coleoptera, Curculionidae, Leptomias Faust. It is mainly distributed in Bayi District, Linzhi, Tibet, China. The genus *Leptomias* comprises 93 species of which 58 are distributed over the Tibet district of the Himalayan region, 19 over other counties or districts of the Himalayan region, and 16 are dispersed over regions or counties other than the Himalayan region (Chao and Chen 1980). More than 70% of the species are distributed between 3000 and 5000 m above sea level (Wang et al. 2010). As a typical plateau and alpine species group, *Leptomias* sp. adapts specifically to drastic high-altitude environmental conditions, by developing dark body coloration and small body size. It is an ideal species for study the Plateau Environment Adaptability.

In this study, the complete mitogenome of the *Leptomias* sp. was sequenced for the first time. The specimens were obtained from the training farm of Tibet Agriculture and Animal Husbandry College (N29°40’01.00”; E94°20’15.00”) at an altitude of 2999.2 meters in 2019. Samples have been deposited in Tibet Academy of Agricultural and Animal Husbandry Sciences with accession number: 20190713-24. The total genomic DNA was extracted from the body using a traditional phenol–chloroform method (Sambrook and Russell 2001). After DNA isolation, 1 μg of purified DNA was fragmented and used to construct short-insert libraries (insert size 430 bp) according to the manufacturer’s instructions (Illumina), then sequenced on the Illumina Hiseq 4000 (Borgstrom et al. 2011). The mitochondria genome was reconstructed using a combination of de novo and reference-guided assemblies, and the following three steps were used to assemble mitochondria genomes. First, the filtered reads were assembled into contigs using SOAPdenovo2.04 (Li et al. 2010). Second, contigs were aligned to the reference genome of species using BLAST, and aligned contigs (≥80% similarity and query coverage) were ordered according to the reference genome. Third, clean reads were mapped to the assembled draft mitochondria genome to correct the wrong bases, and the gaps were filled through local assembly. The tRNA genes were identified and then the secondary structures of tRNAs were predicted using tRNAscan-se (Lowe and Chan 2016). MEGA 7.0 software was used for constructing a Neighbour-Joining (NJ) tree (Kumar et al. 2016).

We obtained a 16,801 bp long complete circular mitochondrial genome of *Leptomias* sp. and submitted in the GenBank (accession no. MT536938). It included 22 transfer RNA genes, 13 protein-coding genes (PCGs), two ribosomal RNA genes, and one control region. The order and orientation of the functional areas of the *Leptomias* sp. mitogenome are similar to those in the *Naupactus xanthographus* and *Sympiezomias velatus* mitogenome (Vera and Bergmann 2018; Zhou 2020). Base frequency of the whole mtDNA region was A = 38.7%, T = 34.3%, C = 27.0%, and G = 10.0%, the content of A + T in the complete genome was 73.0%. Most of the genes (nine PCGs and 14 tRNAs) were encoded on the majority strand (J-strand), whereas other genes (four PCGs, two rRNAs, and eight tRNAs) were encoded on the minority strand (N-strand). All of the 22 tRNAs ranging from 63 bp (trnG and trnH) to 71 bp (trnK) and Tween-one tRNA gene were found to be a typical secondary cloverleaf structure, except for trnS1 which lacked the dihydrouridine arm did not form a stable structure. The rrnL was 1056 bp long and the rrnS was 792 bp long.

One newly generated mitogenomes and 18 from GenBank were analyzed in this study.

The phylogenetic tree (Figure 1) was constructed using the maximum-likelihood methods by MEGA 7. We observed that *Leptomias* sp. was closely related to *Sympiezomias velatus* and *Sciobius* sp. than other species. In conclusion, our study of mitogenome of *Leptomias* sp. will provide important valuable data for the future research in molecular identification and mitochondrial inheritance mechanism. Therefore, more mitogenomes need to be sequenced in further studies.

**Figure 1.**
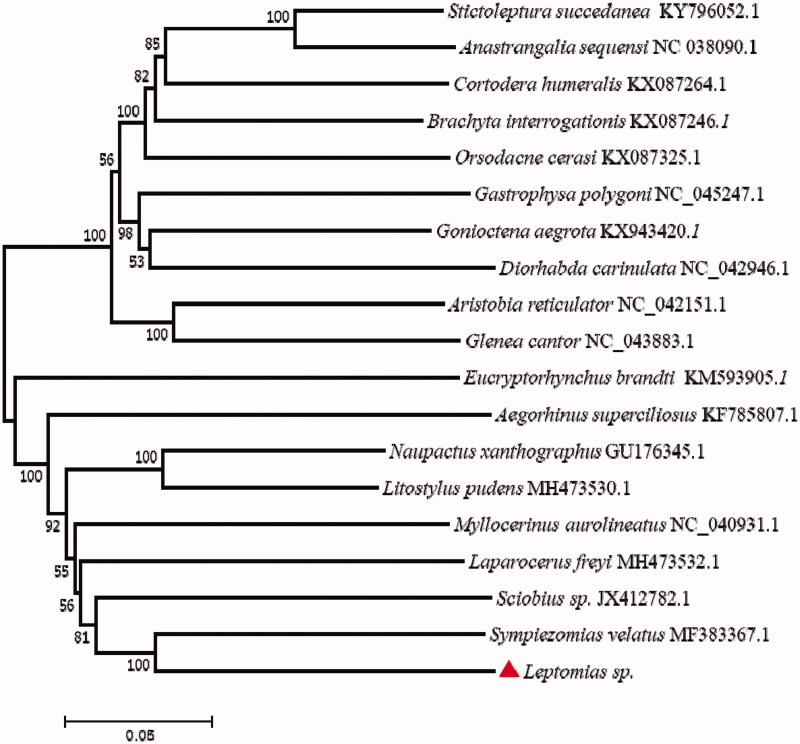
The consensus phylogenetic relationship of the *Leptomias* sp. (accession no. MT536938) with other 18 species. Phylogenetic tree based on the complete mitochondrial genome sequences was constructed using neighbour-joining method. GenBank accession numbers of mitogenomic sequences for each taxon are shown in parentheses.

## Data Availability

The data that support the findings of this study are openly available in GenBank of NCBI at https://www.ncbi.nlm.nih.gov, reference number MT536938
